# Supplementary material for: A mitofusin-dependent docking ring complex triggers mitochondrial fusion in vitro
Source: eLife. 2016 Jun 2;5:e14618. doi: 10.7554/eLife.14618 (PMC4929004; doi:10.7554/eLife.14618)
Supplement: Supplementary file 1. — DOI: http://dx.doi.org/10.7554/eLife.14618.026 [file elife-14618-supp1.docx]

**Supplementary file 1**

| **Name** | **Back-ground** | **Genotype** | **occurrence in the study** |
| --- | --- | --- | --- |
| W303 WT (MCY553) | W303 | *Mata ura3-1 trp1-1 leu2-3,112 his3-11,15 can1-100 RAD5 ADE2* | 1B; 2A; 2B; 3A; 3B; 4A-D; 5A; 5B; 6A; 6B |
| W303 + Fzo1 o.e. (MCY1273) | W303 | *Mata FZO1::LEU2 ura3-1 trp1-1 leu2-3,112 his3-11,15 can1-100 RAD5 ADE2 +* *pRS414-TEF-FZO1* | 7E; 7F |
| W303 Fzo1 (MCY1154) | W303 | *Matα FZO1::LEU2 ura3-1 trp1-1 leu2-3,112 his3-11,15 can1-100 RAD5 ADE2 +* pRS314-FZO1 | 7A; 7B; 7D |
| DF5 Mito-mCherry (#980) | DF5 | *Mata his3-Δ200, leu2-3,2-11::TEF-Mito-mCherry:LEU2, lys2-801, trp1-1, ura3-52* | 1C, 6D |
| DF5 OM45-GFP (#779) | DF5 | *Mata OM45-GFP::KanMX4, his3-Δ200, leu2-3, lys2-801, trp1-1(am), ura3-52* | 1C , 6D |
| W303 *fzo1Δ* (MCY620) | W303 | *Matα FZO1::LEU2 ura3-1 trp1-1 leu2-3,112 his3-11,15 can1-100 RAD5 ADE2 + PRS314* | 7A; 7B |
| W303 Fzo1 o.e. (MCY1222) | W303 | *Matα FZO1::LEU2 ura3-1 trp1-1 leu2-3,112 his3-11,15 can1-100 RAD5 ADE2 +p414-TEF-Fzo1* | 7A; 7B; 7D |
| W303 Fzo1 mito-GFP (MCY810) | W303 | *Matα FZO1::LEU2 ura3-1 trp1-1 leu2-3,112 his3-11,15 can1-100 RAD5 ADE2 +* pRS314-FZO1 *+ p426-TEF-mito-GFP* | 7C |
| W303 Fzo1 o.e. mito-GFP (MCY1223) | W303 | *Matα FZO1::LEU2 ura3-1 trp1-1 leu2-3,112 his3-11,15 can1-100 RAD5 ADE2 +p414-TEF-Fzo1+ p426-TEF-mito-GFP* | 7C |
| W303 Fzo1 (MCY1154) | W303 | *Matα FZO1::LEU2 ura3-1 trp1-1 leu2-3,112 his3-11,15 can1-100 RAD5 ADE2 +* pRS314-FZO1 | 8C |
| W303 Fzo1-AviTag (MCY1155) | W303 | *Matα FZO1::LEU2 ura3-1 trp1-1 leu2-3,112 his3-11,15 can1-100 RAD5 ADE2 +pFzo1-AviTag-PRS314* | 8D |

**Table of Strains Used in the Study.**

| **Name (Collection number)** | **Description** | **Reference** |
| --- | --- | --- |
| pRS314 (MC219) | CEN,*TRP1*, Amp | ([Sikorski & Hieter, 1989](#_ENREF_43)) |
| pRS314-FZO1 (MC250) | CEN, *FZO1* promoter-*FZO1*, *TRP1*, Amp | ([Cohen et al, 2011](#_ENREF_9)) |
| p426-TEF-mitoGFP (MC244) | 2 micron, *TEF1* promoter- *Su9*-*GFP*, *URA3*, Amp | This study |
| pRS314-FZO1-AviTag (MC369) | CEN, *TEF1* promoter-*FZO1-Avi*, *TRP1*, Amp | This study |
| p426-TEF_mito-mCherry (MC248) | 2 micron, *TEF1* promoter- *Su9*- mCherry, *URA3*, Amp | This study |

**Table of Plasmids Used in the Study.**
